# Genes Involved in Systemic and Arterial Bed Dependent Atherosclerosis - Tampere Vascular Study

**DOI:** 10.1371/journal.pone.0033787

**Published:** 2012-04-11

**Authors:** Mari Levula, Niku Oksala, Nina Airla, Rainer Zeitlin, Juha-Pekka Salenius, Otso Järvinen, Maarit Venermo, Teemu Partio, Jukka Saarinen, Taija Somppi, VeliPekka Suominen, Jyrki Virkkunen, Juha Hautalahti, Reijo Laaksonen, Mika Kähönen, Ari Mennander, Leena Kytömäki, Juhani T. Soini, Jyrki Parkkinen, Markku Pelto-Huikko, Terho Lehtimäki

**Affiliations:** 1 Department of Clinical Chemistry, Centre for Laboratory Medicine, Tampere University Hospital and Department of Clinical Chemistry, Medical School, University of Tampere, Tampere, Finland; 2 Department of Surgery, Division of Vascular Surgery, Tampere University Hospital, Tampere, Finland; 3 Department of Cardiac Surgery, Heart Center, Tampere University Hospital, Tampere, Finland; 4 Department of Clinical Physiology, University of Tampere and Tampere University Hospital, Tampere, Finland; 5 Turku Center for Biotechnology, Turku, Finland; 6 Department of Pathology, Centre for Laboratory Medicine, Tampere, Finland; 7 Department of Developmental Biology, Medical School, University of Tampere and Department of Pathology, Tampere University Hospital, Tampere, Finland; Heart Center Munich, Germany

## Abstract

**Background:**

Atherosclerosis is a complex disease with hundreds of genes influencing its progression. In addition, the phenotype of the disease varies significantly depending on the arterial bed.

**Methodology/Principal Findings:**

We characterized the genes generally involved in human advanced atherosclerotic (AHA type V–VI) plaques in carotid and femoral arteries as well as aortas from 24 subjects of Tampere Vascular study and compared the results to non-atherosclerotic internal thoracic arteries (n=6) using genome-wide expression array and QRT-PCR. In addition we determined genes that were typical for each arterial plaque studied. To gain a comprehensive insight into the pathologic processes in the plaques we also analyzed pathways and gene sets dysregulated in this disease using gene set enrichment analysis (GSEA). According to the selection criteria used (>3.0 fold change and p-value <0.05), 235 genes were up-regulated and 68 genes down-regulated in the carotid plaques, 242 genes up-regulated and 116 down-regulated in the femoral plaques and 256 genes up-regulated and 49 genes down-regulated in the aortic plaques. Nine genes were found to be specifically induced predominantly in aortic plaques, e.g., lactoferrin, and three genes in femoral plaques, e.g., chondroadherin, whereas no gene was found to be specific for carotid plaques. In pathway analysis, a total of 28 pathways or gene sets were found to be significantly dysregulated in atherosclerotic plaques (false discovery rate [FDR] <0.25).

**Conclusions:**

This study describes comprehensively the gene expression changes that generally prevail in human atherosclerotic plaques. In addition, site specific genes induced only in femoral or aortic plaques were found, reflecting that atherosclerotic process has unique features in different vascular beds.

## Introduction

Atherosclerosis is a complex disease characterized by endothelial cell dysfunction, smooth muscle cell proliferation and migration, inflammation, lipid and matrix accumulation and thrombus formation with hundreds of genes influencing its progression. Susceptibility to atherosclerosis is in turn influenced by complex gene-gene and gene-environment interactions making atherosclerosis a challenging research subject.

Gene expression techniques, such as microarrays and representational difference analysis, are powerful tools that can be used to discover the complexities underlying the development of atherosclerotic plaque. This method has previously been used to detect differentially expressed genes in normal and diseased arteries [1], [2], disease progression [3], detecting differentially expressed genes according to patient symptomatology [4] and discovering pathways affected in coronary atherosclerosis [5]. When considering the large amount of genes influencing the development of atherosclerosis focusing into pathway characterization provides a comprehensive insight about the pathological mechanisms underlying atherosclerosis. On the other hand, single-gene approach may be utilized when analyzing fundamental genes in complex signalling systems.

Although atherosclerosis has a systemic nature, the susceptibility to develop atherosclerotic lesions and the histological type of atherosclerosis differs strikingly between different sites in human vasculature. The type of atherosclerosis ranges from stable calcified plaques and fibrotic plaques all the way to unstable ulcerated plaques and the prevalence of these lesions varies according to vascular bed region. For example, ulcerated plaques in symptomatic carotid stenosis patients are common while fibrotic and calcified lesions dominate in aortic and femoral areas raising the question whether this dissimilarity could also be seen in the gene expression profiles in different vascular regions.

We, therefore, screened the global gene expression profile of advanced atherosclerotic plaques in carotid arteries, femoral arteries and aortas and compared the results to non-atherosclerotic left internal thoracic arteries (LITA) and identified most up- and down-regulated genes in each arterial bed and searched for genes that would be specific for one arterial region, and in addition, characterized genes that were generally involved in disease. Using gene set enrichment analysis (GSEA), we also analyzed pathways (available in MSigDB database) that were generally affected in atherosclerotic plaques.

## Methods

### Tampere Vascular Study (TVS) material

The atherosclerotic vascular sample series for GWEA consists of atherosclerotic plaques from the following arterial sites: femoral artery (n=4) carotid artery (n=9) and abdominal aorta (n=7) and control samples from internal thoracic arteries (ITA) during coronary artery bypass surgery (n=6) all together from a total of 26 patients participating in Tampere Vascular Study. All the samples were handled and obtained in a standardized fashion supervised by senior scientist in our laboratory. All the samples from atherosclerotic arteries were obtained by endarterectomy under loupe magnification obtaining a sample that consists of the plaque with intima and the inner media. All these procedures were performed by vascular surgeons under the surveillance of one of the principal investigators (NO). ITA samples consisted of arterial rings obtained during dissection and with all the arterial layers including outer media and adventitia. All the patients had a polyvascular disease (i.e. at least two major arterial beds affected by atherosclerotic plaques as evidenced by 1) previous transient ischemic attack and/or atherosclerotic plaques in the cerebral vasculature or 2) coronary atherosclerosis as evidenced by previous myocardial infarction or 3) angina pectoris and atherosclerotic plaques in coronary angiography or 4) objectively verified peripheral arterial disease by ankle-brachial pressure index <0.9 or 5) previous arterial surgery due to atherosclerosis or 6) angiographical demonstration of arterial plaques. Of these patients, only two had polyvascular disease and all the rest had monovascular disease limited to the coronary vasculature. The sample population demographics are presented as Table S1. The population had strong male predominace. The aortic group were the youngest and had seldom dyslipidemia and diabetes. The control group had more seldom hypertension and diabetes. The cholesterol levels were highest in femoral and control group. Smoking was frequent, especially in aortic group.

For the relative gene expression analysis, 24 atherosclerotic tissue samples were used (2 from the original sample set could not be recovered) and similarly, the six ITA vessels were used as controls. The vascular samples were histologically classified according to the American Heart Association classification (AHA) [6]. The carotid and femoral artery samples were type V or VI, aorta samples were type VΙ and all control vessels were healthy. The study was approved by the Ethics Committee of Tampere University Hospital (Permission number 99204). All the patients gave written informed consent. The samples were taken from patients subjected to open vascular surgical procedures at the Division of Vascular Surgery, Tampere University Hospital. All the patients gave informed consent.

### RNA isolation and genome wide expression analysis

The fresh tissue samples were soaked in RNALater solution (Ambion Inc., Austin, TX, USA) and isolated with Trizol reagent (Invitrogen, Carlsbad, CA, USA) and the RNAEasy Kit (Qiagen, Valencia, CA, USA). The concentration and quality of the RNA was evaluated spectrophotometrically (BioPhotometer, Eppendorf, Wesseling-Berzdorf, Germany). More than 23,000 known and candidate genes were analyzed using Sentrix Human-8 Expression BeadChips, according to manufacturer's instructions. (Illumina, San Diego, CA, USA). In brief, a 200 ng aliquot of total RNA from each sample was amplified to cDNA using the Ambion's Illumina RNA Amplification kit according to the instructions (Ambion, Inc., Austin, TX, USA). Each sample cRNA (1500 ng) was hybridized to Illumina's Sentrix Human-8 Expression BeadChip arrays (Illumina). Hybridized biotinylated cRNA was detected with 1 µg/ml Cyanine3-streptavidine (Amersham Biosciences, Pistacataway, NJ, USA). BeadChips were scanned with the Illumina BeadArray Reader. The method has been described in more detail in our previous work [7].

### Bioinformatics and statistical analyses

The data was archived using the minimum information about a microarray experiment (MIAME 1.1. Draft 6). Raw intensity data obtained from the IlluminaTM platform were normalized with R language and environment for statistical computing and related Bioconductor module [8]. Bioconductor module was also used to conduct single-probe analysis including fold-change calculations and filtering the probes. The statistical analysis was carried out using the Limma package [9]. -Pathway analysis of the expression data (all diseased vs. controls) was performed using the GSEA implemented in GSEA java desktop application version 2.0 and MsigDB (Molecular Signature Database) version 2.0. Statistical analysis was performed using SPSS version 14.0. (SPSS Inc., Chicago, IL, USA). The non-parametric Mann-Whitney U-test with post-hoc correction was used for comparison of mRNA expression between atherosclerotic and control tissues and to find differentially expressed genes. The results are presented as average fold change. The averaging was done for each arterial bed. The selection criteria were >3.0-fold change in gene expression and p-value less than 0.05. The agreement between GWEA and TLDA was evaluated by first classifying the results as down-regulated, neutral or up-regulated. Then the number of samples correctly classified into these categories was calculated and was found to be >90%.

### Quantitative RT-PCR

Quantitative gene expression analyses were performed with TaqMan low density arrays (TLDAs) (Applied Biosystems, Foster City, CA, USA) using gene specific TaqMan gene expression assays. Total-RNA (500 ng) was transcribed to cDNA using the High Capacity cDNA Kit (Applied Biosystems) according to manufacturer's instructions. After the cDNA synthesis, the LDA cards were loaded with 8 µl undiluted cDNA, 42 µl H_2_0, and 50 µl PCR Universal Master Mix (Applied Biosystems) and run according to the manufacturer's instructions. Samples were analyzed as duplicates, and both cDNA synthesis and PCR reactions were validated for inhibition of amplification in PCR and cDNA synthesis. Glyceraldehyde 3-phosphate dehydrogenase (GAPDH) was used as a housekeeping gene. The results were analyzed using SDS 2.2 Software (Applied Biosystems).

### Immunohistochemistry

All samples were first dyed with haematoxyclin (HE) and classified according to Stary et al. [6]. Immunocytochemistry was performed using the N-Histofine® Simple Stain MAX PO staining method (Nichirei Biosciences Inc., Tokyo, Japan)) and paraffin-embedded vascular samples without any counterstain. Lactotransferrin (LTF)- immunoreactivity(IR) was detected with a rabbit polyclonal antibody (dil. 1∶100, Lifespan Bioscience, Seattle, WA, USA). Vascular cell types were identified with mouse anti-human muscle actin (dil. 1∶30, clone HHF35; DakoCytomation, Glostrup, Denmark), mouse anti-human endothelial cell (dil. 1∶70, CD31, clone JC70A; DakoCytomation) and mouse anti-human CD68 (dil. 1∶70, clone PG-M1, DakoCytomation) was used as marker of monocytes and macrophages. Neutrophil granulocytes were identified using mouse anti-human neutrophil elastase antibody (dil. 1∶500, DakoCytomation). T-lymphocytes were recognized with mouse anti-CD3 antibody (dil. 1∶150, eBioscience Inc., San Diego, CA) and B-lymphocytes were labelled with mouse anti-CD20 (dil. 1∶1000, DakoCytomation. The sections were subjected to microwave antigen retrieval treatment as described earlier [10] except for elastase antibody. Endogenous peroxidase activity was extinguished by treating the section with with 0.3% H_2_O_2_ for 30 min. Subsequently the sections were incubated overnight with the primary antibodies followed with appropriate *N*-Histofine staining reagent for 30 min. ImmPACT™ (Vector Laboratories, Burlingame, CA, USA) diaminobenzidine-solution with nickel-intensification was used as the chromogen. All antibodies were diluted in PBS containing 1% BSA and 0.3% of Triton X-100. Controls included omitting the primary antibody or replacing it with non-immune sera. No staining was seen in the controls. The co-localization of LTF with different markers was studied in adjacent 5 µm sections (mirror image sections). Sections were stained as described above. Photographs were obtained using Nikon FXA-100 microscope equipped with PCO Sensicam digital camera (PCO, Kelheim, Germany).

## Results

### Overall gene expression changes observed in carotid, femoral and aortic plaques

Several genes were found to have significantly altered expression in advanced atherosclerotic carotid and femoral artery plaques as well as in the aortas studied with GWEA. According to the selection criteria used (>3.0 fold change and p-value <0.05), 235 genes were up-regulated and 68 genes down-regulated in type V–VI carotid plaques. For type V–VI femoral plaques, 242 genes were up-regulated and 116 genes down-regulated. In type VI aortic plaques, 256 genes were up-regulated and 49 genes down-regulated (Table S2, S3, S4, S5, S6, S7). In order to identify globally affected genes, we combined all gene expression results and calculated average fold changes. Of these, 27 most up-regulated and 16 down-regulated genes were verified with QRT-PCR (TLDA) (Table 1 and 2). The concordance between GWEA and TLDA was over 90%.

**Table 1:** The most up-regulated genes generally in atherosclerotic plaques analysed with TaqMan Low Density array.

*Gene abbreviation*	*Gene ID*	*Average FC*
MMP12 (matrix metallopeptidase 12)	4321	473.7 (p<0.0001)
MMP7 (matrix metallopeptidase 7)	4316	686.1 (p<0.0001)
SPP1 (secreted phosphoprotein 1)	6696	173.0 (p<0.0001)
APOC1 (apolipoprotein C–I)	341	154.8 (p<0.001)
MMP9 (matrix metallopeptidase 9)	4318	125.3 (p<0.001)
CCL18 (CC chemokine ligand 18)	6362	105.7 (p<0.001)
ACP5 (acid phosphatase 5)	54	57.7 (p<0.001)
APOE (apolipoprotein E)	348	52.9 (p<0.001)
IL4I1 (interleukin 4 induced 1)	259307	35.2 (p<0.001)
RGS1 (regulator of G-protein signaling 1)	5996	19.9 (p<0.001)
HMOX1 (heme oxygenase 1)	3162	20.5 (p<0.001)
IFI30 (interferon, gamma-inducible protein 30)	10437	23.2 (p<0.001)
SLAMF8 (SLAM family member 8)	56833	22.0 (p<0.001)
MGC29506 (hypothetical protein)	51237	19.2 (p=0.001)
THBS1 (thrombospondin 1)	7057	13.8 (p=0.000)
LYZ (lysozyme)	4069	10.8 (p<0.001)
IGJ (immunoglobulin J polypeptide)	3512	14.1 (p=0.001)
TYMP (thymidine phosphorylase)	1890	14.0 (p<0.001)
IL8 (interleukin 8)	3576	12.0 (p<0.001)
COL1A1 (collagen, type I, alpha 1)	1277	5.7 (p<0.001)
CAPG (capping protein)	822	7.4 (p<0.001)
ADFB (adipose differentiation-related protein)	123	6.4 (p<0.001)
LGALS3 (lectin, galactoside-binding, soluble, 3)	3958	1.8 (p=0.001)
CYBA (cytochrome b-245, alpha polypeptide)	1535	5.4 (p<0.001)
CFL1 (cofilin 1)	1072	1.3 (p=0.158)
ALDOA (aldolase A, fructose-bisphosphate)	226	1.2 (p<0.001)
HLA-DRP3 (major histocompatibility complex, class II, DR beta 3)	3125	−8.8 (p=0.561)

The results are shown as an average fold change (FC) compared to control arteries.

**Table 2:** The most generally down-regulated genes in atherosclerotic plaques analysed with TaqMan Low Density array.

*Gene abbreviation*	*Gene ID*	*Average FC*
ITLN1 (intelectin 1)	55600	−568.2 (p<0.001)
APOD (apolipoprotein D)	347	−103.9 (p<0.001)
DUSP26 (dual specificity phosphatase 26)	78986	−21.3 (p<0.001)
CASQ2 (calsequestrin 2)	845	−23.6 (p<0.001)
RGS5 (regulator of G-protein signaling 5)	8490	−13.0 (p<0.001)
TCEAL2 (transcription elongation factor A (SII)-like 2)	140597	−18.6 (p<0.001)
DES (desmin)	1674	−15.8 (p=0.025)
CALD1 (caldesmon 1)	800	−10.4 (p<0.001)
PPP1R3C (protein phosphatase 1) regulatory (inhibitor) subunit 3C)	5507	−9.5 (p<0.001)
ADRA2C (adrenergic, alpha-2C-, receptor)	152	−12.9 (p<0.001)
CSRP2 (cysteine and glycine-rich protein 2)	1466	−8.6 (p<0.001)
SPEG (SPEG complex locus)	10290	−4.2 (p<0.001)
CNN1 (calponin 1)	1264	−4.3 (p<0.001)
C6orf117 (chromosome 6 open reading frame 117)	112609	−8.7 (p<0.001)
LMOD1 (leiomodin 1)	25802	−6.5 (p<0.001)
RAMP1 (receptor activity modifying protein 1)	10267	−5.4 (p<0.001)

The results are shown as an average fold change (FC) compared to control arteries.

Among the most up-regulated genes verified with QRT-PCR in atherosclerotic plaque (Table 1), we found genes already previously connected to atherosclerosis, like matrix metalloproteinases [11], apolipoproteins [12] and osteopontin [13], but we also found new genes, not found to be involved in the pathogenesis of atherosclerosis, namely interleukin 4 induced 1 (IL4I1), interferon, gamma-inducible protein (IFI30), SLAM family member 8 (SLAMF8), and immunoglobulin J polypeptide (IGJ). Previously, gene expression profiling of human atherosclerotic coronary arteries did not reveal the involvement of these genes in the pathogenesis on atherosclerosis [14], [5].

We quantitated the 16 most generally down-regulated genes in advanced atherosclerotic plaques using QRT-PCR. For most of the genes on this list there are only few studies in the literature and no information about their connection to atherosclerosis. The significantly down-regulated genes in all the plaques studied are shown in Table 2.

### Site-specific gene expression alterations in different arterial beds

**Figure 1:**
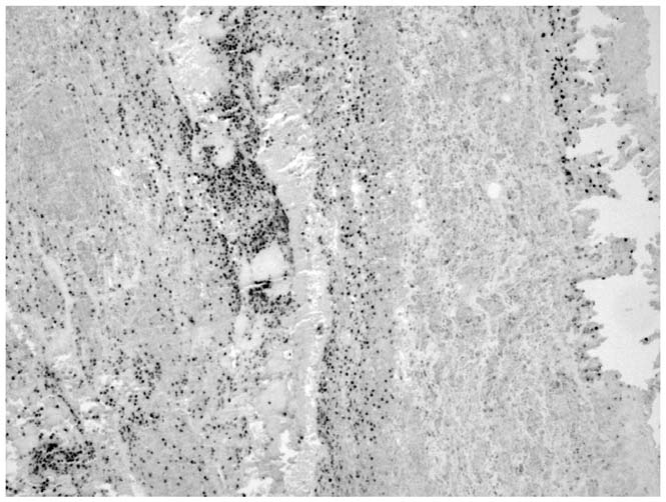
Immunohistochemical staining of lactoferrin (LTF) in advanced human atherosclerotic aorta. The picture was taken with 100× magnitude.

**Figure 2:**
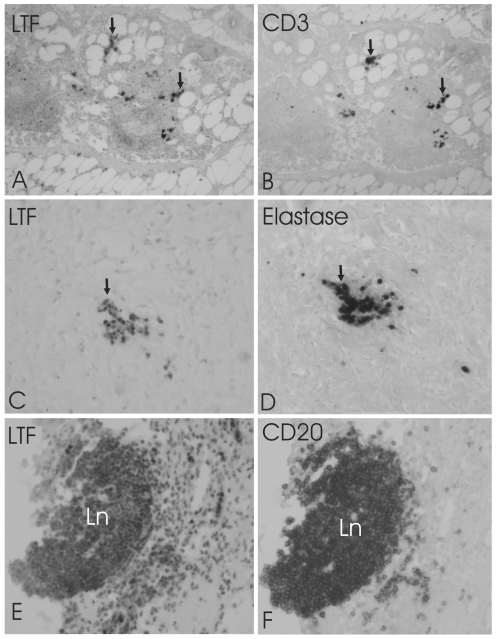
Co-localization of lactoferrin (LTF) with neutrophils and T and B lymphocytes. Adjacent mirror image sections demonstration the co-localization of LTF (A, C, E) with T-cell marker CD3 (B), neutrophil granulocyte marker elastase (D) and B-cell marker CD20 (F) in aortic plaques. Arrows point cells that are both LTF and CD3-IR (A, B) or LTF and Elastane-IR (C, D). Most of the CD20-IR cells in a lymph nodule (Ln) are also LTF-IR. Large number of LTF-IR cells in surrounding tissue are not CD20-IR. The pictures were taken with 200× magnitude.

**Figure 3:**
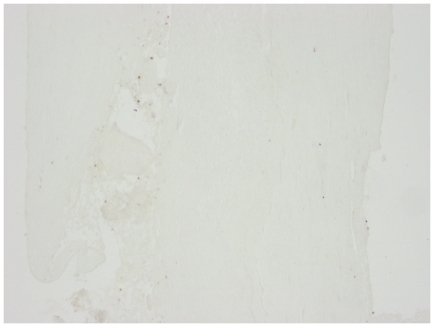
Immunohistochemical staining of lactoferrin (LTF) in advanced human atherosclerotic femoral artery. The picture was taken with 100× magnitude.

**Figure 4:**
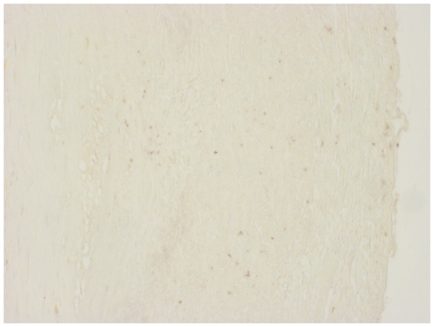
Immunohistochemical staining of lactoferrin (LTF) in advanced human atherosclerotic carotid artery. The picture was taken with 100× magnitude.

According to GWEA data, we found nine genes that were induced only in aortic plaques and three genes that were specifically induced in femoral plaques of which expression was verified with QRT-PCR. Genes induced predominantly in aortic plaques differed considerably from the genes induced in femoral plaques. Most of the genes induced in aortic plaques are involved in immunological processes, especially involving B cells whereas the genes induce in femoral plaques function mainly in extra-cellular matrix modifications (Table 3). The genes that were induced in carotid arteries, were also induced in aortic and femoral plaques, thus no specific gene for carotid plaques was found. To verify the predominant expression in only one arterial bed region, we ascertained the protein localization of one of the aortic plaque specific genes; LTF, a major immune system modulator [15]. Using IHC, LTF protein was found predominantly in aortic plaques mainly in necrotic debris in intima and inner media wherein localized to neutrophils, B and T lymphocytes (Figures 1 and 2). In femoral and carotid arteries, LTF was present only in sparse cells being mostly unoccupied by LTF positive cells (Figures 3 and 4).

**Table 3:** Genes that were induced only in aortic or femoral plaques compared to non-atherosclerotic internal thoracic arteries analyzed with genome-wide expression array (GWEA).

*Gene (ID)*	*Gene ID*	*Fold change*	*Function*
***Aortic plaques***			
CHGA, chromogranin A	1113	5.8	Secretory granules formation, immunity against microbes [66], cancer [67], hypertension and myocardial infarction [38], [68]
CSF3, colony stimulating factor 3	1440	4.6	Survival/proliferation of neutrophils and macrophages [69], arteriogenesis [70]
GAGE12I, G antigen 12I	26748	4.5	Antigen, anti-apoptotic factor [71], [72]
C4orf7, choromosome 4 open reading frame 7	260436	4.3	B cell immunity, tumorigenesis [73]
GAGE6, G antigen 6	2578	4.0	Antigen, cancer [74]
LTF, lactotransferrin	4057	3.7	Immune modulator [15]
PRPH, peripherin	5630	3.6	Intermediate filament [75]
MS4A1, membrane-spanning 4-domains	931	3.5	B cell immunity [76], [77], cancer [78]
***Femoral plaques***			
C1QTNF3, C1q and tumor necrosis factor related protein 3	114899	3.4	Adipose tissue secreted protein, anti-inflammatory [79]
CHAD, chondroadherin	1101	3.9	Extracellular matrix structure modification [42], [80]
PTN, pleiotrophin	5764	4.7	Cell differentiation [48], angiogenesis [47], cancer [81]

For all genes, the p-value was less than 0.05.

### Altered pathways in advanced atherosclerotic lesions

In order to identify globally affected pathways in advanced atherosclerosis, we performed gene set expression analyses (GSEA) to illuminate dysregulated pathways. In the pathway analyses, 20 pathways appeared to be significantly up-regulated and 8 pathways down-regulated in advanced atherosclerotic plaques as compared to non-atherosclerotic arteries according to the criteria recommended by Subramanian et al. (FDR<0.25) [16] (Table S8). Significantly up-regulated pathways involved apoptotic and pro-inflammatory pathways as well as pathways involved in complement or B cell activation and cell movement. The significantly altered down-regulated pathways included fatty acid metabolism and amino acid metabolism pathways, glutamate receptor pathway, benzoate degradation pathway and pathway including genes of hormonal functions. Interestingly, a pathway including HOX genes related to hematopoiesis was significantly down-regulated.

Despite intensive research on the role of T cells in atherogenesis, this is the first time that all the major genes involved in T cell differentiation are described from three major atherosclerotic arterial beds. In order to verify the results of the pathway analysis, we quantitated with QRT-PCR the expression of all genes belonging to nkTPathway (Biocarta) (natural killer T-cells) containing genes involved in T cell differentiation. The pathway included a total of 29 genes of which 26 were significantly up-regulated and three genes down-regulated in plaques vs. non-atherosclerotic arteries (Table 4). In general, the highest fold changes were seen in aortas (for exact fold changes in different vascular beds, see Table S9). Despite the classical T cell genes already known to be activated, several other genes were also significantly altered in plaques, e.g., chemokine receptors 3,4, and 7 (CCR3, CCR4, CCR7), interferon gamma receptor 1–2 (IFNGR1, IFNGR2), interleukin 12 receptor beta 1–2 (IL12RB1, IL12RB2), interleukin 18 receptor 1 (IL18R1), interleukin 4 receptor (IL4R), and transforming growth factor beta 2 (TGFB2).

**Table 4:** The expression of nkTPathway (natural killer T-cell) genes in atherosclerotic plaques from carotid arteries, aortas and femoral arteries compared to non-atherosclerotic internal thoracic arteries analyzed with TaqMan low density array.

*Gene abbreviation*	*Gene ID*	*Average FC*
CSF2 (colony stimulating factor 2 (granulocyte-macrophage)	1437	↑↑↑*
IL12B (interleukin 12B)	3593	↑↑*
CCR5 (chemokine (C-C motif) receptor 5)	1234	14.9 (p<0.001)
CXCR4 (chemokine (C-X-C motif) receptor 4)	7852	12.4 (p=0.001)
CCR1 (chemokine (C-C motif) receptor 1)	1230	10.7 (p<0.001)
CD4 (CD4 molecule)	920	10.2 (p<0.001)
CCL4 (chemokine (C-C motif) ligand 4)	6351	8.4 (p=0.001)
CCR7 (chemokine (C-C motif) receptor 7)	1236	8.1 (p=0.001)
CCL3 (chemokine (C-C motif) ligand 3)	6348	6.6 (p<0.001)
IL12RB1 (interleukin 12 receptor, beta 1)	3594	6.0 (p<0.001)
CD28 (CD28 molecule)	940	5.8 (p=0.001)
CXCR3 (chemokine (C-X-C motif) receptor 3)	2833	5.6 (p=0.001)
CCR4 (chemokine (C-C motif) receptor 4)	1233	3.5 (p=0.033)
CD40LG (CD40 ligand)	959	3.4 (p=0.008)
IFNG (interferon, gamma)	3458	3.1 (p=0.022)
IL12RB2 (interleukin 12 receptor, beta 2)	3595	3.0 (p=0.233)
IFNGR2 (interferon gamma receptor 2)	3460	2.9 (p<0.001)
IL12A (interleukin 12A)	3592	2.9 (p=0.009)
TGFB1 (transforming growth factor, beta 1)	7040	2.0 (p<0.001)
IL18R1 (interleukin 18 receptor 1)	8809	2.0 (p=0.007)
CCR2 (chemokine (C-C motif) receptor 2)	1231	1.9 (p=0.213)
IFNGR1 (interferon gamma receptor 1)	3459	1.7 (p=0.005)
IL4R (interleukin 4 receptor)	3566	1.4 (p=0.002)
TGFB2 (transforming growth factor, beta 2)	7042	1.4 (p=0.069)
IL5 (interleukin 5)	3567	1.5 (p=0.500)
IL4 (interleukin 4)	3565	−1.2 (p=0.468)
IL2 (interleukin 2)	3558	−2.1(p=0.836)
TGFB3 (transforming growth factor, beta 3)	7043	1.2 (p=0.350)
CCR3 (chemokine (C-C motif) receptor 3)	1232	−1.7 (p=0.108)

Fold changes (FC) are calculated by comparing the median expression of genes in atherosclerotic arteries vs. controls. Notes.

* Highly expressed in atherosclerotic plaque.

## Discussion

The present scan of all known human genes in the atherosclerotic plaques from carotid and femoral arteries as well as from the aortas revealed novel genes involved generally in atherosclerosis and specifically in different vascular regions. In addition, we revealed pathways and gene sets that were significantly dysregulated in advanced atherosclerotic plaques and verified the expression of all genes in one of the most up-regulated pathways that is involved in T cell differentiation.

Many of the genes found in this study have been linked to atherosclerosis also in previous studies. Although, due to rapidly evolved whole genome microarray technology, it is challenging to directly compare the results with those of the previous studies [2], [1], [5], [3]. Furthermore, these studies have utilized samples at different stage of atherosclerosis from organ donors and autopsies [2], [1], [3] whereas pathway analysis has been done focusing only in the coronary region in the heart transplantation patients [5].

### New genes involved in advanced atherosclerosis

In addition to genes already linked to atherosclerosis, we also found a new set of genes, not previously found in atherosclerotic plaques, or even otherwise connected to the disease. IL4I1 (FC+35.2), is expressed by B-cells, [17], [18] and in inflammatory conditions by macrophages and dendritic cells [19]. Interferon, gamma-inducible protein 30 (IFI30) (FC+23.2) demonstrated high expression and it has not been previously identified in atherosclerosis. Since both IL41I [19], and IFI30 [20], [21] participate in the down-regulation of Th1 mediated inflammation, a crucial element in atherosclerosis [22], [23], our results of high expression of IL41I and IFI30 may reflect a negative feedback response to Th1 mediated inflammation in the atherosclerotic process. Our results of the involvement of IFI30 and IFI30 in the regulation of Th1 cells opens new possibilities to modulate the immune reactions responsible for atherogenic processes. It is also interesting to note that even though some immunoglobulins, like IgG and IgM [24], [25], have already been linked to atherosclerosis, there is no information in the literature about IgJ, involved in B cell activation [26], in atherosclerosis. Based on the NCBI Gene Expression Omnibus database, IgJ has had elevated expression in some of the samples in gene expression profiling of atherosclerotic coronary arteries, but the results were controversial and did not support a critical role in atherosclerosis [5]. In our study though, the expression of IgJ in all three atherosclerotic arteries was obvious with 14.1-fold up-regulation in plaques and with p-value of p<0.001 warranting further studies.

We found several significantly up-regulated genes in plaques without obvious implications in atherosclerosis. These genes include e.g., CCL18 and RGS1, involved in lymphocyte attraction, migration and function [27]–[29] and CAPG that is suggested to modulate the protective effects of unidirectional shear stress [30]. BLAME, a B cell costimulator and adhesion molecule [31] that has previously found in human macrophages to be induced in response to LDL [32] and now, found +22.0 –fold in human advanced atherosclerotic plaques. Most of the significantly down-regulated genes are new with regards to atherosclerosis. The most drastically down-regulated gene was intelectin 1 (ITLN1), which was almost absent in atherosclerotic plaques compared to non atherosclerotic internal thoraci`c arteries. ITLN1 is a cell surface phagocytotic receptor that recognizes specific bacterial cell wall components [33] and the absence of ITLN1 has been suggested to alter immune responses to infection and facilitate inflammation [34]. Another significantly down-regulated gene was the regulator of G-protein signalling 5 (RGS5). Recently, the blockage of RGS5 has been suggested to provide an alternative approach to treat hypertension but the biological impact of the reduced expression of RGS5 in the plaques is not known.

### Site-specific gene expression changes in vascular regions studied

After carefully examining the GWEA data, we determined nine genes specific for aortic plaques and three genes specific for femoral plaques. We did not find any gene that would have been specific for carotid plaques. It is interesting to note that the genes that were specifically induced in aortic plaques are mostly involved in immune reactions, especially in B cell immunity (Table 3).

We observed a significant up-regulation of LTF in aorta, located in B- and T-cells and in neutrophils. LTF is an iron-binding glycoprotein abundantly found in exocrine secretions of mammals and released by mucosal epithelia and neutrophils during inflammation [35] and binds to cells of the immune system [15].

The role of LTF as a negative regulator of inflammation, is a key element in the host defence system and is capable of binding to cells of the immune systems, e.g., cells of the monocyte lineage [15]. LTF interacts with monocytes and macrophages and modulates their function during inflammatory and infectious processes, e.g., increasing cytotoxic activity, cytokine production (Th1) and expression of surface molecules [36]. When considering the fundamental role of macrophages in atherosclerosis, it is interesting to speculate the reason why such a powerful macrophage regulator is induced predominantly in aortic plaques. Interestingly, serum lactoferrin has been found to associate with fatal ischemic heart disease in patients with diabetes [37]. Whether the LTF expression induce specific immunological effects in aortic plaques contributing to phenotype commonly observed in aortic plaques, needs to be clarified in the future. At least, considering the wide array of immunological functions, such speculation is justifiable.

Chromogranin A (CHGA) that acts as a prohormone giving rise to several biologically active peptides [38], was another gene found to be specific for aortic plaques. CHGA has been suggested to be a fundamental regulator of blood pressure [38] and endothelial barrier function [39] and interestingly, an independent predictor of long-term mortality and heart failure of acute coronary syndromes [40]. The biological role of CHGA in the pathogenesis of atherosclerosis is not known but the ∼6-fold up-regulation specifically in aortic plaques may inspire future research.

The three genes induced specifically in femoral plaques differed from the aortic plaque specific genes. Femoral plaques exist usually in a stable phenotype with a placid fibrous cap and therefore it is not surprising that chondroadherin, a cartilage matrix protein, was ∼4-fold induced predominantly in femoral plaques. In addition of being able to promote attachement of osteoblastic cells to solid state substrates [41], CHAD interacts with integrin alpha2beta1 (α2β1) [42] and complement [43], both involved in atherogenic processes [44]–[46].

Pleiotrophin (PTN) was ∼5-fold up-regulated in femoral plaques, has previously been found in atherosclerotic human coronary arteries and suggested to participate in intraplaque vascularization, inflammation [47] and monocyte/macrophage differentiation into endothelial cell phenotype [48].

### T cell differentiation pathway in atherosclerotic plaques

We found the pathway including genes involved in the regulation of T cell chemokine pathway to be highly activated in the advanced plaques. Taken the fundamental role of T cells in the plaque formation and inflammation [49], we quantitated all the genes belonging to this pathway in order to get a comprehensive view about the extent how all the crucial T cell chemokines are actually expressed in human plaques and even more, see the trends of the pathway. Instead of focusing on well-known genes involved in T cell activation, we discuss here genes with little or no previous information at all regarding atherosclerosis.

IL12, secreted by macrophages dendritic cells and Th1 cells and acting through IL12RB1/2 [50], is considered a proatherogenic and proinflammatory cytokine contributing to chemotaxis and migration [23], [51]. IL12RB1 was 6-fold up-regulated in the plaques and IL12RB2 3-fold, respectively. It is interesting to speculate that the dissimilar expression of the two subunits of IL12R may alter the consequences of IL12 binding and even may have distinct effects of their own in the plaque immunological reactions. Actually, IL12RB2 has already found to limit cancer growth and thus proven to elicit an effect on its own [52].

IL2 and IL4 were the only interleukins on this pathway that were generally down-regulated in plaques. Previously, pro-inflammatory IL2 has been found to be associated with carotid artery intima-media thickness [53]. Locally produced IL4 has earlier expected to be protective towards an excessive pro-inflammatory response in plaques [54], [55] but there are also studies that suggest a pro-atherogenic role for IL4 [56]. Considering the low expression of IL2 and IL4 mRNA in atherosclerotic plaques, the role of these cytokines may not be significant in atherosclerosis. It is though worth on mentioning that the receptor for IL4, IL4R, was actually up-regulated in plaques. In addition of binding IL4, IL4R is also capable of binding IL13, a cytokine with potential anti-inflammatory activity [57] raising the idea whether the role of IL4R should be investigated in more detail with regards inhibiting the pathologic processes leading to atherosclerotic disease.

TGFB1–3 genes were all only mildly up-regulated in plaques studied. A study by Mallat et al. with apoE-deifcient mice suggest a major protective role for TGFB signaling in atherosclerosis. In this study, inhibition of TGFB signaling was also found to induce an unstable plaque phenotype [58]. The protective role seemed to depend on the effects of TGFB on macrophages and T cells, major players in atherosclerosis pathogenesis. As the expression of TGFB1–3 was only mildly up-regulated in plaques, the inducement of TGFB expression or signaling might offer new strategies in preventing the plaque progression.

With the exception of CCR2 and CCR3, all the other CCRs in this pathway were significantly up-regulated in plaques with fold changes varying from 14.9 to 3.5 supporting the previously suggested role of CCRs in this disease [59], [60]. CCR3, a marker for Th2 cells, showed significant down-regulation (fold change −4.5, p=0.012) only in carotid arteries suggesting that the Th2 response in carotid artery disease may differ from aortic and femoral artery atherosclerosis.

Interferon gamma has been suggested to accelerate atherosclerosis e.g. by activating macrophages and increasing their production of nitric oxide and pro-inflammatory cytokines [61]. In our study, the expression of interferon gamma receptors 1 and 2 (IFNGR1, IFNGR2) was significantly up-regulated in all atherosclerotic arteries studied. Previously, IFNGR1 and −2 have been found in carotid artery atheroma [62] but their role in the disease is not known. Its is though interesting to note that IFNGR1 acts as an major player in Th1 cell differentiation [63] and that IL4 has been found to prevent the association of IFNGRs in the antigen recognition [64]. In this study, the IL4 mRNA was low in atherosclerotic plaques. Whether the low IL4 expression could impact the function IFNGRs, remains to be evaluated in the future. In addition, it remains to be seen in the future whether the INFG mediated actions in atherosclerotic plaques could be modulated through manipulation of its receptors.

### Limitations of the study

In this study, non atherosclerotic internal thoracic arteries (ITA) were used as control vessels in gene expression analysis. Even though comparison of the gene expression between healthy and diseased vessels from the same origin would have given more accurate results, unfortunately we were not able to obtain any healthy samples from the carotid or femoral arteries nor from the aortas due to ethical issues. We still believe that ITA vessels as controls provide valuable information for discovering the pathological biological processes going on in atherosclerotic plaques. It must be emphasized that the differences in hydrostatic pressure component in between the carotid and femoral territory, vascular compliance, and the differences in flow velocity in aorta compared with either carotid or femoral territory, result in huge variation in endothelial shear stress and artery wall radial load potentially modulating the gene expression. However, the present data still provides valuable information about the mechanism that hemodynamics may affect the development of atherosclerosis although the significance of this factor cannot be separately addressed in the present experimental setting. The gene expression profile may also be affected by differences in the cell type composition in different arterial beds which has not been characterized in the present study. A laser micro-dissection with appropriate mRNA quantitation method would provide cell specific information about the expression of genes. This technique has also previously been successfully applied to gene expression studies with atherosclerotic arteries [2], [65]. Another shortcoming is that the control vessels contained outer media and adventitia not present in atherosclerotic plaque samples. This approach may enrich the cell types present only in the intima and inner media of diseased vessels and thereby affecting gene expression results. Since the macrophages are the dominating type of inflammatory cells, the changes in macrophage related genes are also pronounced. It must be emphasized that the different sample groups were not adjusted for gender, age, lipid parameters or medication. At the present, the study population consisted of rather old (median age 70.0 years) subjects with a male predominance, majority having hypertension and history of smoking. This limits the generalization of the present results as the present data is limited to a high risk populations with severe symptoms and signs of atherosclerosis.

### Conclusions

Atherosclerosis is a complex disease with numerous factors influencing disease development and phenotype. We analyzed advanced human atherosclerotic carotid and femoral artery plaques as well as aortas and screened all genes that were dysregulated in these arteries. In addition, we evaluated all the pathways and gene sets that were differentially expressed in diseased vessels. The study represents comprehensively the gene expression changes prevailing in three major advanced arterial beds and suggests several new, previously unknown genes to be involved in the disease pathogenesis. As the three major arterial beds; carotid, femoral, and aortic plaques were thoroughly studied in this study, the results serve also as an excellent reference for further studies in the future.

## Supporting Information

Table S1 **Demographics of sample population.**
(DOC)

Table S2 **Significantly up-regulated genes in human advanced atherosclerotic aorta.**
(XLS)

Table S3 **Significantly down-regulated genes in human advanced atherosclerotic aorta.**
(XLS)

Table S4 **Significantly up-regulated genes in human advanced atherosclerotic femoral artery.**
(XLS)

Table S5 **Significantly down-regulated genes in human advanced atherosclerotic femoral artery.**
(XLS)

Table S6 **Significantly up-regulated genes in human advanced atherosclerotic carotid artery.**
(XLS)

Table S7 **Significantly down-regulated genes in human advanced atherosclerotic carotid artery.**
(XLS)

Table S8 **Significantly altered pathways in advanced atherosclerotic plaques from arteries analyzed with gene set enrichment analysis (GSEA). All pathways are found in the MSigDB database.**
(DOC)

Table S9 **The expression of nkTPathway genes in atherosclerotic plaques from carotid arteries, aortas and femoral arteries analyzed with TaqMan Low Density array.** Fold changes are calculated by comparing the median expression of genes in atherosclerotic arteries vs. controls. Genes marked boldface represent a specific expression pattern dependable upon arterial bed.
(DOC)
