# Supplementary material for: Genes Involved in Systemic and Arterial Bed Dependent Atherosclerosis - Tampere Vascular Study
Source: PLoS One. 2012 Apr 11;7(4):e33787. doi: 10.1371/journal.pone.0033787 (PMC3324479; doi:10.1371/journal.pone.0033787)
Supplement: Table S1 — Demographics of sample population. (DOC) [file pone.0033787.s001.doc]

Table S1. Demographics of sample population

|  | Carotid artery  (n=9) | Femoral artery  (n=6) | Aorta  (n=7) | Internal thoracic artery  (ITA) (n=6) |
| --- | --- | --- | --- | --- |
| Male gender  Age, mean (S.D.)  Height, mean (S.D.)  Weight, mean (S.D.)  Dyslipidemia (%)  Hypertension (%)  Diabetes (%)  fS-Chol, mean (S.D.)  Smoking (%) | 5/9 (56.0%)  72.4 (8.7)  166.8 (11.7)  76.4 (14.0)  4/9 (44.4)  7/9 (77.8)  2/9 (22.2)  5.0 (0.14)  6/9 (66.7) | 6/6 (100%)  72.2 (11.0)  177.7 (10.4)  80.2 (8.6)  3/6 (50.0)  6/6 (100.0)  2/6 (33.3)  7.0 (0.0)  5/6 (83.3) | 7/7 (100%)  63.4 (12.6)  176.1 (4.6)  78.0 (9.8)  1/7 (14.3)  5/7 (71.4)  1/7 (14.2)  6.1 (0.1)  7/7 (100.0) | 6/6 (100%)  72.2 (10.5)  177.7 (6.7)  80.2 (11.8)  3/6 (50.0)  4/6 (66.7)  1/6 (16.7)  7 (1.4)  4/6 (66.7) |
