# Supplementary material for: Genes Involved in Systemic and Arterial Bed Dependent Atherosclerosis - Tampere Vascular Study
Source: PLoS One. 2012 Apr 11;7(4):e33787. doi: 10.1371/journal.pone.0033787 (PMC3324479; doi:10.1371/journal.pone.0033787)
Supplement: Table S8 — Significantly altered pathways in advanced atherosclerotic plaques from arteries analyzed with gene set enrichment analysis (GSEA). All pathways are found in the MSigDB database. (DOC) [file pone.0033787.s008.doc]

Table S8. Significantly altered pathways in advanced atherosclerotic plaques from arteries analyzed with gene set enrichment analysis (GSEA). All pathways are found in the MSigDB database.

| ***Pathway name*** | ***Number of genes*** | ***FDR-value**** | Source | Description |
| --- | --- | --- | --- | --- |
| *Up-regulated pathways (n=20)* |  |  |  |  |
| tnfr2Pathway | 18 | 0.239 | BioCarta | TNFB binds to TNFRS to induce activation in immune cells and apoptosis |
| nktPathway | 29 | 0.241 | BioCarta | T cell differentiation into Th1 and Th2 by differential chemokine receptor expression |
| cell_motility | 118 | 0.243 | Gene Ontology | Cell movement |
| raccycdPathway | 23 | 0.243 | BioCarta | G1/S transition |
| Fas signaling pathway | 9 | 0.244 | Sigma-Aldrich | Fas induced apoptosis |
| ST_Phosphoinositide_3_Kinase_Pathway | 38 | 0.245 | Signaling Transduction KE | Regulation of cell growth, survival and movement |
| salmonellaPathway | 12 | 0.245 | BioCarta | Mechanism of salmonella to enter cells |
| caspasePathway | 23 | 0.246 | BioCarta | Apoptotic signaling |
| hivnefPathway | 58 | 0.246 | BioCarta | Apoptotic signaling |
| HOGERKORP_ANTI_CD44_DN | 12 | 0.246 | Broad Institute | Genes differentially expressed in B cells |
| MAP_KINASE_KINASE_KINASE_  ACTIVITY | 10 | 0.246 | Gene Ontology | MAP kinase pathway |
| Complement_Activation_Classical | 16 | 0.246 | GenMAPP | Complement activation cascade |
| il1rPathway | 33 | 0.246 | BioCarta | IL1R1 mediated IL1 response |
| stressPathway | 25 | 0.247 | BioCarta | TNF/Stress related signaling |
| deathPathway | 33 | 0.247 | BioCarta | Apoptotic signaling |
| ST_B_Cell_Antigen_Receptor | 40 | 0.248 | Signaling Transduction KE | B cell receptors and activation |
| JECHLINGER_EMT_UP | 57 | 0.248 | Broad Institute | Genes upregulated for epithelial plasticity in tumor progression |
| il5Pathway | 10 | 0.248 | BioCarta | IL5 signaling |
| Par1Pathway | 21 | 0.249 | BioCarta | Thrombin signaling and protease-activated receptors |
| SA_B_CELL_RECEPTOR_COMPLEXES | 24 | 0.249 | Sigma-Aldrich | B cell receptor mediated signaling |
| *Down-regulated pathways (n=8)* |  |  |  |  |
| betaoxidationPathway | 6 | 0.125 | MgSigDB | Beta-oxidation of fatty acids |
| GPCRs_Class_C_Metabotropic_glutamate_  pheromone | 14 | 0.125 | Gene Ontology | Glutamate receptor pathway |
| MAP00632_Benzoate_degradation | 10 | 0.155 | GenMAPP | Degradation of benzoate |
| MAP00650_Butanoate_metabolism | 29 | 0.161 | GenMAPP | Butanoate metabolism |
| MAP00280_Valine_leucine_and_isoleucine_  degradation | 36 | 0.163 | GenMAPP | Degradation of leucine and isoleucine |
| BRENTANI_HORMONAL_FUNCTION | 12 | 0.199 | Broad Institute | Cancer related genes in hormonal functions |
| HOX_LIST_JP | 56 | 0.205 | Broad Institute | HOX genes related to hematopoiesis |
| MAP00071_Fatty_acid_metabolism | 47 | 0.243 | GenMAPP | Fatty acid metabolism |

Abbreviations: FDR; false discovery rate

Statistics: * FDR < 0.25 was considered significant according to the criteria recommended by Subramanian et al (11).
