# Supplementary material for: Genes Involved in Systemic and Arterial Bed Dependent Atherosclerosis - Tampere Vascular Study
Source: PLoS One. 2012 Apr 11;7(4):e33787. doi: 10.1371/journal.pone.0033787 (PMC3324479; doi:10.1371/journal.pone.0033787)
Supplement: Table S9 — The expression of nkTPathway genes in atherosclerotic plaques from carotid arteries, aortas and femoral arteries analyzed with TaqMan Low Density array. Fold changes are calculated by comparing the median expression of genes in atherosclerotic arteries vs. controls. Genes marked boldface represent a specific expression pattern dependable upon arterial bed. (DOC) [file pone.0033787.s009.doc]

Table S9. The expression of nkTPathway genes in atherosclerotic plaques from carotid arteries, aortas and femoral arteries analyzed with TaqMan Low Density array. Fold changes are calculated by comparing the median expression of genes in atherosclerotic arteries vs. controls. Genes marked boldface represent a specific expression pattern dependable upon arterial bed.

| ***Gene abbreviation*** | ***Gene ID*** | ***FC in carotid arteries*** | ***FC in aortas*** | ***FC in femoral arteries*** | ***Average FC*** |
| --- | --- | --- | --- | --- | --- |
| **CSF2 (colony stimulating factor 2 (granulocyte-macrophage)** | 1437 | ↑↑↑* | ↑↑↑* | ↑↑↑* |  |
| **IL12B (interleukin 12B)** | 3593 | ↑↑* | ↑↑* | ↑↑* |  |
| **CCR5 (chemokine receptor 5)** | 1234 | 14.9 (p= 0.000) | 20.3 (p= 0.002) | 9.4 (p= 0.009) | 14.9 (p=0.000) |
| **CXCR4 (chemokine receptor 4)** | 7852 | 4.6 (p=0.000) | 24.1 (0.000) | 8.5 (p= 0.041) | 12.4 (p= 0.001) |
| **CCR1 (chemokine receptor 1)** | 1230 | 13.6 (p= 0.000) | 11.1 (p= 0.000) | 7.5 (p= 0.002) | 10.7 (p= 0.000) |
| **CD4 (CD4 molecule)** | 920 | 11.3 (p= 0.000) | 12.4 (p= 0.002) | 6.9 (p= 0.002) | 10.2 (p= 0.000) |
| **CCL4 (chemokine ligand 4)** | 6351 | 5.4 (p= 0.008) | 10.6 (p= 0.000) | 9.1 (p= 0.009) | 8.4 (p= 0.001) |
| **CCR7 (chemokine receptor 7)** | 1236 | 4.6 (p=0.005) | 15.9 (0.002) | 3.9 (p= 0.026) | 8.1 (p= 0.001) |
| **CCL3 (chemokine ligand 3)** | 6348 | 5.7 (p=0.002) | 8.0 (p= 0.000) | 6.2 (p= 0.002) | 6.6 (p= 0.000) |
| **IL12RB1 (interleukin 12 receptor, beta 1)** | 3594 | 5.6 (p=0.002) | 8.8 (p= 0.002) | 3.7 (p= 0.002) | 6.0 (p= 0.000) |
| **CD28 (CD28 molecule)** | 940 | 6.5 (p= 0.000) | 7.3 (p= 0.000) | 3.5 (p= 0.065) | 5.8 (p= 0.001) |
| **CXCR3 (chemokine receptor 3)** | 2833 | 3.5 (p= 0.002) | 9.7 (p= 0.008) | 3.6 (p= 0.009) | 5.6 (p= 0.001) |
| **CCR4 (chemokine receptor 4)** | 1233 | 1.7 (p= 0.328) | **5.5 (p= 0.008)** | 3.2 (p= 0.132) | 3.5 (p= 0.033) |
| **CD40LG (CD40 ligand)** | 959 | 2.1 (p= 0.036) | **5.9 (p= 0.008)** | 2.3 (p= 0.093) | 3.4 (p= 0.008) |
| **IFNG (interferon, gamma)** | 3458 | 1.4 (p= 0.776) | **5.9 (p= 0.000)** | 1.9 (p= 0.026) | 3.1 (p= 0.022) |
| **IL12RB2 (interleukin 12 receptor, beta 2)** | 3595 | 1.3 (p= 0.529) | **2.6 (p= 0.088)** | 1.0 (p= 0.818) | 3.0 (p= 0.233) |
| **IFNGR2 (interferon gamma receptor 2)** | 3460 | 2.7 (p= 0.002) | 3.5 (p= 0.000) | 2.5 (p= 0.002) | 2.9 (p=0.000) |
| **IL12A (interleukin 12A)** | 3592 | 2.1 (p= 0.113) | **4.8 (p= 0.003)** | 1.7 (p= 0.065) | 2.9 (p= 0.009) |
| **TGFB1 (transforming growth factor, beta 1)** | 7040 | 2.0 (p= 0.000) | 2.1 (p= 0.000) | 1.8 (0.009) | 2.0 (p= 0.000) |
| **IL18R1 (interleukin 18 receptor 1)** | 8809 | 1.3 (p= 0.050) | **3.4 (p= 0.000)** | 1.3 ( 0.240) | 2.0 (p= 0.007) |
| **CCR2 (chemokine receptor 2)** | 1231 | 1.6 (0.864) | **2.8 (p= 0.012)** | 1.4 (p= 0.937) | 1.9 (p= 0.213) |
| **IFNGR1 (interferon gamma receptor 1)** | 3459 | 1.6 (p= 0.012) | **2.2 (p= 0.000)** | 1.4 (p= 0.394) | 1.7 (p= 0.005) |
| **IL4R (interleukin 4 receptor)** | 3566 | 1.1 (p= 0.776) | **1.8 (p= 0.018)** | 1.3 (0.041) | 1.4 (p= 0.002) |
| **TGFB2 (transforming growth factor, beta 2)** | 7042 | - 1.4 (p= 0.113) | **- 1.4 (p= 0.088)** | - 1.3 (0.394) | 1.4 (p= 0.069) |
| **IL5 (interleukin 5)** | 3567 | - 1.8 (p= 0.272) | 1.1 (p= 0.776) | 1.3 (p= 1.000) | 1.5 (p= 0.500) |
| **IL4 (interleukin 4)** | 3565 | - 2.5 (p= 0.066) | - 1.1 (p= 0.864) | 2.1 (0.589) | - 1.2 (p= 0.468) |
| **IL2 (interleukin 2)** | 3558 | - 1.7 (p= 0.018) | **2.5 (p= 0.008)** | - 1.2 (0.394) | - 1.2 (p= 0.836) |
| **TGFB3 (transforming growth factor, beta 3)** | 7043 | **- 2.7 (p= 0.001)** | 1.3 (p= 0.388) | 1.2 (p= 1.000) | 1.2 (p= 0.350) |
| **CCR3 (chemokine receptor 3)** | 1232 | **- 4.5 (p= 0.012)** | - 1.6 (p= 0.145) | 1.1 (p= 0.699) | - 1.7 (p=0.108) |

Note. *; highly expressed in atherosclerotic plaque
